# Induced Tauopathy in a Novel 3D-Culture Model Mediates Neurodegenerative Processes: A Real-Time Study on Biochips

**DOI:** 10.1371/journal.pone.0049150

**Published:** 2012-11-07

**Authors:** Diana Seidel, Dana Krinke, Heinz-Georg Jahnke, Anika Hirche, Daniel Kloß, Till G. A. Mack, Frank Striggow, Andrea Robitzki

**Affiliations:** 1 Centre for Biotechnology and Biomedicine (BBZ), University of Leipzig, Division of Molecular Biological-Biochemical Processing Technology, Leipzig, Germany; 2 Translational Centre for Regenerative Medicine, University of Leipzig, Leipzig, Germany; 3 KeyNeurotek Pharmaceuticals AG, Zenit Technologiepark, Magdeburg, Germany; 4 Department of Neurodegeneration and Intervention Strategies, German Center for Neurodegenerative Diseases (DZNE), Magdeburg, Germany; Research Institute of Environmental Medicine, Nagoya University, Japan

## Abstract

Tauopathies including Alzheimer’s disease represent one of the major health problems of aging population worldwide. Therefore, a better understanding of tau-dependent pathologies and consequently, tau-related intervention strategies is highly demanded. In recent years, several tau-focused therapies have been proposed with the aim to stop disease progression. However, to develop efficient active pharmaceutical ingredients for the broad treatment of Alzheimer’s disease patients, further improvements are necessary for understanding the detailed neurodegenerative processes as well as the mechanism and side effects of potential active pharmaceutical ingredients (API) in the neuronal system. In this context, there is a lack of suitable complex *in vitro* cell culture models recapitulating major aspects of taupathological degenerative processes in sufficient time and reproducible manner.

Herewith, we describe a novel 3D SH-SY5Y cell-based, tauopathy model that shows advanced characteristics of matured neurons in comparison to monolayer cultures without the need of artificial differentiation promoting agents. Moreover, the recombinant expression of a novel highly pathologic fourfold mutated human tau variant lead to a fast and emphasized degeneration of neuritic processes. The neurodegenerative effects could be analyzed in real time and with high sensitivity using our unique microcavity array-based impedance spectroscopy measurement system. We were able to quantify a time- and concentration-dependent relative impedance decrease when Alzheimer’s disease-like tau pathology was induced in the neuronal 3D cell culture model. In combination with the collected optical information, the degenerative processes within each 3D-culture could be monitored and analyzed. More strikingly, tau-specific regenerative effects caused by tau-focused active pharmaceutical ingredients could be quantitatively monitored by impedance spectroscopy.

Bringing together our novel complex 3D cell culture taupathology model and our microcavity array-based impedimetric measurement system, we provide a powerful tool for the label-free investigation of tau-related pathology processes as well as the high content analysis of potential active pharmaceutical ingredient candidates.

## Introduction

Today, more than 35 million patients are suffering from dementia including Alzheimeŕs disease (AD) [1] and there are estimations that the number of AD cases will further rise to more than 100 million in 2050 [2], [3]. Therefore, a detailed understanding of the underlying mechanisms leading to disease onset and progression is essential to develop novel therapeutic strategies. Extracellular β amyloid (Aβ) plaques and intracellular tau fibrils are the two hallmarks of AD pathology. In the past, the majority of AD-related active pharmaceutical ingredients (API) development programs were focused on Aβ. More recently, accumulating evidence has suggested that both, Aβ and tau might be involved in the initiation, manifestation and progression of AD [4], [5], [6]. However, the molecular mechanisms that may link Aβ- and tau-induced signaling cascades and underlie AD are still not completely known. An insufficient molecular understanding of AD pathology itself as well as inappropriate preclinical disease and screening models are likely a substantial cause for many failures in clinical active pharmaceutical ingredients (API) development in AD over the past years [7].

In this context, we have developed a novel 3D *in vitro* cell culture model, which is based on the human neuroblastoma cell line SH-SY5Y that have been previously used in several tau pathology studies [8], [9], [10]. However, the use of spheroid cultures, instead of monolayer cultures, might be preferred, since 3D cultures recapitulate the *in vivo* situation likely better than 2D cell cultures [11], [12]. Especially in the field of neurodegenerative diseases, neuronal differentiation, neurite formation and spatial orientation in tissue-like cultures are crucial for the development of an AD-like pathology [13]. To recapitulate the AD-related tau pathology, we generated three SH-SY5Y cell lines that stably overexpress N-terminal EGFP-fused human tau (0N4R) variants: the wildtype (WT), the single mutated tau variant P301L and a novel fourfold mutated variant comprising the single point mutations ΔK280, P301L, V337M, R406W. These mutations have been identified in frontotemporal dementia and parkinsonism-linked to chromosome 17 (FTDP-17) related taupathology and are widely used to recapitulate AD-like tau pathology in *in vivo* models [14]. In contrast to methods like lipofection, calcium phosphate precipitation or even adenoviral transduction the lentiviral transduction of the WT and mutated 4R tau variants ensures a homogenous and moderate stable expression of transgenic tau with a 10–15 fold increased expression level in comparison to native SH-SY5Y cells and an expression level comparable to primary neuronal *in vitro* cultures [15], [16]. To achieve a reproducible tau-specific pathology in an appropriate time scale of several days, we further used the selective protein phosphatase 2A inhibitor okadaic acid (OA) that is commonly used to induce tau hyperphosphorylation *in vitro* [8], [17] and *in vivo* [18].

Despite the advantages of more complex 3D culture models, there are only limited possibilities for sensitive quantifications of cellular alterations. Especially in the context of label-free real time monitoring for the establishment of standardized screening assays, we developed an electrical impedance spectroscopy-based (EIS) read-out system [8], [19] that, in combination with a unique microcavity array [16], [20], results in an outstanding tool for the investigations of pathologic processes and moreover, for screening of potential APIs. For demonstrating the performance of our system, we used reference compounds that have previously shown therapeutic benefits in mice models [21], [22]. In detail, we investigated the efficacy of a specific (AR-A014418) and a non-specific GSK-3β inhibitor (hymenialdisine) and the HSP-90 inhibitor 17-allylamino-17-demothoxygeldanamycin (17-AAG), which had proven to prevent tau hyperphosphorylation [23], [24], [25], as well as the tau aggregation inhibitor methylene blue that was evaluated in a phase II trial [26].

## Results

### 3D Cultivation of SH-SY5Y Cells Leads to Increased Neuronal Differentiation

With the objective to establish a 3D *in vitro* model for the label-free monitoring of tauopathy-related degeneration of neuronal cells, especially neuritic processes, that is highly reproducible and feasible for high-content screening systems, we generated SH-SY5Y cell lines stably expressing an N-terminal fused EGFP tau protein by lentiviral transduction. In detail, we established three SH-SY5Y cell lines overexpressing human 0N4R tau variants: wildtype (WT), the common used single point mutation P301L and moreover, a novel fourfold mutation variant K280q comprising the ΔK280, P301L, V337M, R406W mutations. The poly-mutation was generated with the aim to strengthen the tauopathy.

**Figure 1:**
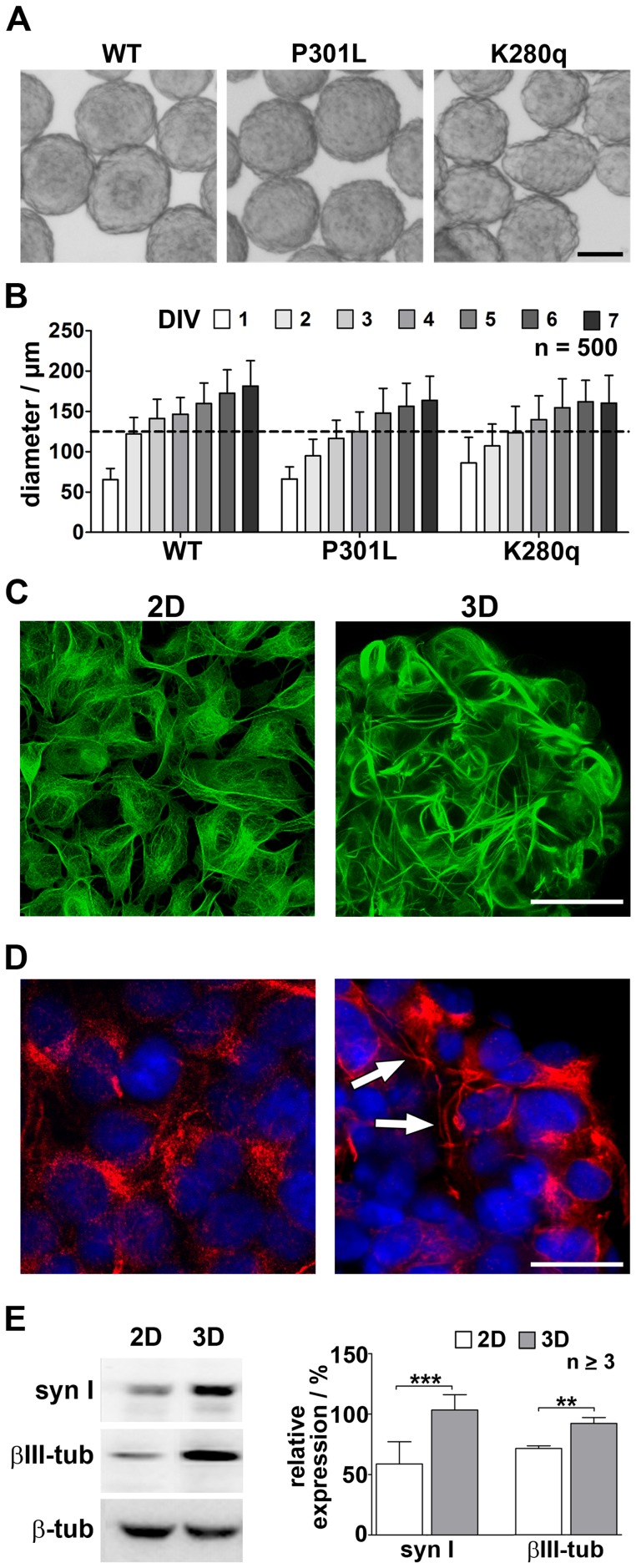
3D cultivation of tau expressing SH-SY5Y cells leads to enhanced neuronal differentiation. A. Stably EGFP-tau fusion protein expressing SH-SY5Y cells were cultivated on a gyratory shaker in order to generate spheroids within the first five days *in vitro* (DIV) (bar = 100 µm). **B.** Statistical analysis of spheroid diameter revealed comparable size development over time for wildtype (WT), single-mutated (P301L) and poly-mutated (K280q) tau expressing SH-SY5Y cells (mean ± s.d.). **C.** Live cell imaging of the EGFP-fused wildtype tau (green) and **D.** immunocytochemical staining of the neuronal marker NF200 (red) ensured the overall existence of matured neural cells and revealed extended neuritic processes (arrows) in five days cultivated spheroids (3D) in comparison to monolayer cultures (2D) (bar = 20 µm, nuclear stain DAPI blue). **E.** The expression of the neuronal markers synapsin I (syn I)and β III-tubulin (βIII-tub) in wildtype tau expressing 2D and 3D cultures at culture day seven were analyzed via Western blot and quantified relative to the housekeeping protein β-tubulin.(n ≥3, **p<0.01, ***p<0.001).

Since SH-SY5Y cells are derived from a neuroblastoma, the neuronal differentiation of monolayer cultures is poorly developed and has to be promoted via retinoic acid, staurosporine or neurotrophic factors as NGF or BDNF [13], [27]. Since differentiation agents were found to affect various signaling pathways, their application would probably influence the tauopathy induction [28], [29]. Thus, we established more organotypic-like spheroid cultures from all three cell lines (Fig. 1A) by using a self-developed gyratory shaker system [30]. The generated spheroids of all cell lines showed a comparable size and shape development over seven days of cultivation (Fig. 1B) and reached an appropriate size for analysis with a diameter preferably greater than 125 µm after four to five days. By visualizing the EGFP-fused wildtype protein in 2D and 3D cultures, it was possible to show a considerably higher number and even elongation of neuronal processes in the 3D cultures (Fig. 1C, Movie S1). To prove the consistent neuronal character of cells within the spheroids, the localization of the neuronal marker NF200 was analyzed by immunocytochemistry. The staining revealed the distribution of neurofilament structures within all cells concentrated in the prolonged neural processes composing a 3D unit but not in the monolayer culture (Fig. 1D). Those findings were supported by Western blot analysis of the neuronal markers synapsin I and β III-tubulin in WT tau expressing cells cultured as monolayers or spheroids. After seven days of cultivation, both neuronal markers showed higher expression in 3D cultures (Fig. 1E). The densitometric analysis revealed an extreme significant increase of synapsin I expression in 3D cultures (107±8%) compared to 2D cultures (59±8%). Consistently, β III-tubulin expression increased in 3D cultures (93±3%) in comparison to 2D cultures (72±1%).

Together, this impressively shows that using 3D culture systems enhances neuronal differentiation at least for SH-SY5Y cells without the need for artificial differentiation agents.

### Tau Hyperphosphorylation Leads to Increased Degenerative Processes in Mutated Tau Expressing 3D Cultures

**Figure 2:**
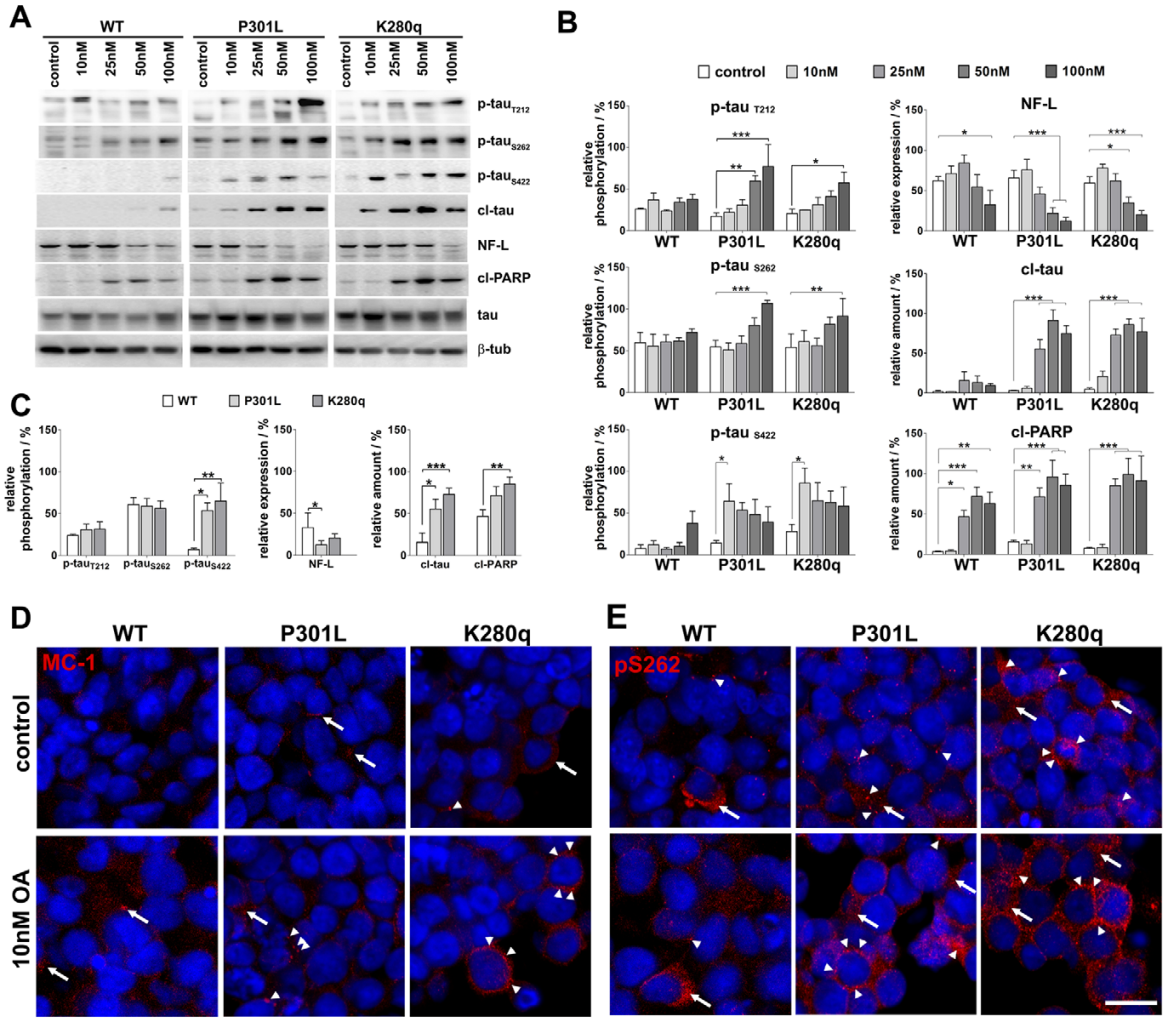
Analysis of cellular alterations after okadaic acid induced hyperphosphorylation in wildtype and mutated tau expressing SH-SY5Y spheroids. **A.** Western blot analysis of five days cultivated WT, P301L and K280q tau expressing SH-SY5Y spheroids after incubation with okadaic acid (OA) for 24 hours. Hyperphosphorylation was proven by determination of the pathological relevant tau epitopes T212 (p-tau_T212_), S262 (p-tau_S262_) and S422 (p-tau_S422_). Cellular degradation was analyzed by the caspase-3 cleavage products tau_D421_ (cl-tau) and cleaved-PARP (cl-PARP) as well as neurofilament-L degradation (NF-L). **B.** The phosphorylation of the three tau epitopes was quantified relative to total tau, NF-L expression and cleaved protein amounts were quantified relative to β-tubulin (β-tub) **C.** Significant changes between the tau variants were analyzed to proof the tau pathology state in the tau mutants. (n = 3, *p<0.05, **p<0.01, ***p<0.001). **D.** For analysis of tauopathy-related alterations in cellular localization and aggregation state of the pathological tau conformation MC-1 (red) and **E.** tau phosphorylated at S262 (red), five days cultivated WT and K280q spheroids were incubated with 10 nM OA for 24 hours. Immunocytochemical staining of untreated WT, P301L and K280q tau expressing spheroids showed the sporadic accumulation (arrows) and aggregation (arrowheads) of pathological microtubule-associated tau protein. Incubation with OA led to a spread accumulation of aggregated MC-1 and p-tau_S262_ tau (arrowheads). Fibrous structures indicate the existence of tau fibrils. (bar = 10 µm, nuclear stain DAPI blue).

To investigate the assumed tau mutation-dependent cellular degeneration, we performed Western blot analysis of tau pathological, axonal and cellular degradation markers. Moreover, we enforced tau pathological processes by incubation with okadaic acid (OA) for 24 hours in a concentration range of 10–100 nM (Fig. 2A). For proofing induced tau hyperphosphorylation, we analyzed the pathological relevant tau epitopes T212, S262 and S422 (Fig. 2B). Furthermore, we compared the named markers between the tau variants for the OA concentration 25 nM (Fig. 2C). Densitometric analysis of three independent experiments revealed that relative phosphorylation of T212 and S262 was comparable in untreated WT (26±1% and 59±12%), P301L (17±4% and 55±8%) and K280q (21±5% and 54±16%) tau expressing cells (Fig. 2B). Furthermore, p-tau_T212_ WT tau expressing cells showed no significant increase. In contrast, P301L and K280q tau expressing cells revealed a significant rise to 77±26% (P301L) and 58±13% (K280q) at 100 nM OA. However, significant differences between the tau variants were not visible for 25 nM OA treatment (Fig. 2C). The pathologic effect was increased for the tau epitope S262 where relative phosphorylation was very significantly increased to 107±4% (P301L) and 91±21% (K280q). Again tau variant comparison showed no significant differences at low OA concentrations. A more pronounced phosphorylation impact could be detected for the tau epitope S422, which exhibited a mutation-dependent phosphorylation level even without OA treatment (WT: 7±5%; P301L: 14±3%; K280q: 28±9%) that reached its significant different maximum for P301L and K280q tau at 10 nM OA (64±21% and 86±18%, respectively) but never for WT tau, even at 100 nM OA (38±15%). Tau variant differences could be determined for the moderate OA concentration 25 nM, the S422 phosphorylation of tau mutants were found to be significant (P301L) and very significant different (K280q) from the WT tau. Interestingly, analysis of the axonal marker protein neurofilament-L (NF-L) showed a slight non-significant increase of expression at lower OA concentrations. Higher concentrations of OA induced a progressive and significant reduction of NF-L expression to 32±17% (WT), 12±4% (P301L) and 20±5% (K280q) implying an advanced neurite and cellular degeneration. More strikingly, significant differences between the tau mutants and the WT at 25 nM could be detected.

Next, we analyzed the amount of caspase 3-cleaved tau. The C-terminal truncation of tau (Asp421) at 25 nM OA was found to be doubled in WT tau expressing spheroids, whereas OA treatment in P301L and K280q tau expressing spheroids led to 15 and 12 times higher amounts compared to the control. In contrast, the analysis of the caspase 3-cleaved amount of PARP (Asp214) showed a similar progression for all three cell lines. However, treatment with 25 nM OA induced an extreme significant increase of cleaved PARP amounts to 46±8% in WT, 71±11% in P301L and 85±9% in K280q 3D cultures. At this concentration, significant differences between the WT tau cell line and the mutants were detectable for both cl-tau and cl-PARP amounts. Interestingly, 10 nM OA was in no case sufficient to significantly change the amounts of these apoptosis markers. However, immunocytochemical stainings showed that the amount of aggregated tau was increased after 24 hour incubation with 10 nM OA (Fig. 2D−E). Especially, the conformational-dependent MC-1 antibody that recognizes a pathological tau conformation [31], [32] revealed low molecular as well as high molecular aggregated tau. While WT tau spheroids exhibited low molecular weight tau accumulation (arrows) especially after treatment with OA, the local aggregation of pathological folded tau was present even in untreated P301L and K280q cells. The incubation with 10 nM OA resulted in high molecular weight tau aggregates in individual cells in a mutation number-dependent pattern (arrowheads). A similar pattern but with increased progression was detected for stained p-tau_S262_ (Fig. 2E). Whereas high molecular weight aggregates (arrows) and low molecular weight aggregates of p-tau_S262_ positive tau (arrowheads) were visible in individual untreated WT tau cells, the number of affected cells and hyperphosphorylated tau deposits was greater for cells with increased mutation number and OA treatment. In the highly pathological K280q tau expressing cells, the application of 10 nM OA for 24 hours was sufficient to induce the formation of p-tau_S262_ deposits in fibrous structures.

Taken together the aggregation patterns of pathological tau, the changed phosphorylation levels of pathological relevant tau epitopes and amounts of NF-L as well as the missing incidence of apoptosis markers up to 25 nM OA proof a selective tauopathy induction in a mutation-dependent manner. However, at higher OA concentrations cytotoxic effects determine the cell fate.

### Highly Sensitive Label-free Monitoring of Tau Mutation-dependent Neurodegeneration by Impedance Spectroscopy

**Figure 3:**
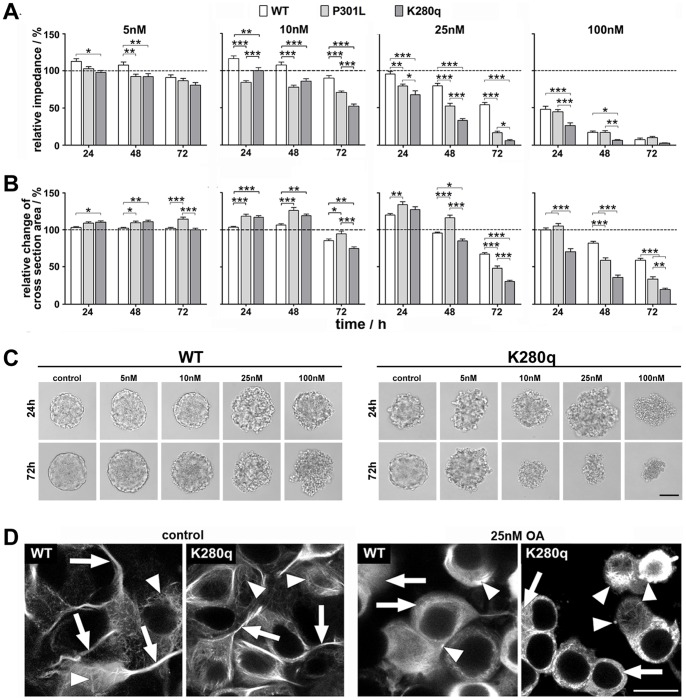
Quantification of tau-specific impedimetric and morphologic alterations induced by okadaic acid treatment. A. SH-SY5Y spheroids expressing WT, P301L and K280q tau were treated with 5 nM, 10 nM, 25 nM and 100 nM okadaic acid (OA) for 24, 48 and 72 hours. Statistical analysis of relative impedance changes normalized to experimental start (100%) and the control revealed tau mutation dependent degeneration of spheroids. **B.** Analysis of cross section area showed a different mutation dependent pattern. (n = 30, *p<0.05, **p<0.01, ***p<0.001) **C.** Example microscopic images of WT and K280q tau expressing spheroids after 24 and 72 hours. (bar = 100 µm) **D.** For analysis of morphological and tau localization alterations, five days cultivated WT and K280q spheroids were incubated with 25 nM OA for 72 hours. Live cell imaging of EGFP-fused untreated WT and K280q tau-expressing spheroids showed the ordered arrangement (arrowheads) of microtubule-associated tau protein especially in the long neuronal processes (arrows). Incubation with OA led to a degeneration of cells and alteration of tau localization (arrows) as well as accumulation (arrowheads) in WT and moreover K280q spheroids. (bar = 50 µm).

After initial molecular and morphological characterization of the induced tau pathological degenerative processes, we wanted to establish a sensitive label-free monitoring system able to quantify the tau mutation-specific effect within a few days. Therefore, we used our microcavity array-based measurement system that was optimized for SH-SY5Y 3D cultures [16]. From the recorded impedance spectra, the maximum cellular contribution was automatically determined in each spectrum and all values were normalized to the measurement start (0 h) and the controls (dashed lines Fig. 3). In total, for each group 30 spheroids from overall three independent experiments were measured every 24 hours (Fig. 3A). Moreover, for each spheroid the cross section area (Fig. 3B) and the morphology (Fig. 3C) were determined by microscopic and live cell images. The relative impedance decreased in a mutation number-, OA concentration- and time-dependent manner. Even after treatment with 5 nM OA for only 24 hours significant differences were observed across WT (113±4%) and K280q (98±3%) tau expressing spheroids. After 72 hours all tau variants showed a relative impedance decrease with a tau mutation number-dependent but not significant effect (WT: 91±3%, P301L: 87±3%, K280q: 81±3%). In contrast, the respective cross section area analysis revealed a mutation number- and time-dependent increase after 48 hours (WT: 102±1%, P301L: 109±2%, K280q: 111±2%). This spheroid cell swelling effect was intensified at 10 nM OA treatment for 24 and 48 hours for the tau mutants. In agreement with this finding, the relative impedance was only decreased in the mutated tau variants with an extreme significant effect (WT: 108±4%, P301L: 78±3%, K280q: 86±3%). The spheroid shape became much more irregular showing blebbed cells. An enhancement of the pathological situation either by prolonged incubation time or a higher OA concentration led to the decrease of both the relative impedance and the cross section area in a significant mutation number-dependent manner. At an OA concentration of 25 nM after a 72 hour incubation period, a strongly declined relative impedance (WT: 54±3%, P301L: 17±2%, K280q: 6±1%) could be correlated with a very low relative cross section area (WT: 67±1%, P301L: 48±3%, K280q: 30±2%) indicating proceeding spheroid degradation. Overall, the application of 100 nM OA led to a much faster relative impedance decrease. Hence, the number of significant impedance differences got smaller with prolonged incubation. Also the increase of cross section area after 24 hours was replaced by a decrease for all tau variants reflecting the degeneration of spheroids to small fragments with progressively shrinking cell structure.

To correlate the documented cell structure alterations with morphological intercellular degenerative processes, live images of SH-SY5Y cells expressing EGFP-fused tau proteins were taken after a 72 hours treatment with OA (Fig. 3D). While in control spheroids the ordered network of microtubule-associated tau (arrowheads) and the presence of long and defined neuronal processes (arrows) were obvious, the incubation with 25 nM OA led to degradation of the neuronal processes as well as the tau dissociation from the microtubule network, visible in a more homogenously distributed cellular background fluorescence. OA-treated WT tau expressing cells still exhibited small processes, which were almost completely lost in K280q tau expressing spheroids. Furthermore, accumulations of EGFP-tau were visible in 3D cultures, especially in K280q tau expressing cells exposed to 25 nM OA, where neurodegeneration was visible (arrowheads).

**Figure 4:**
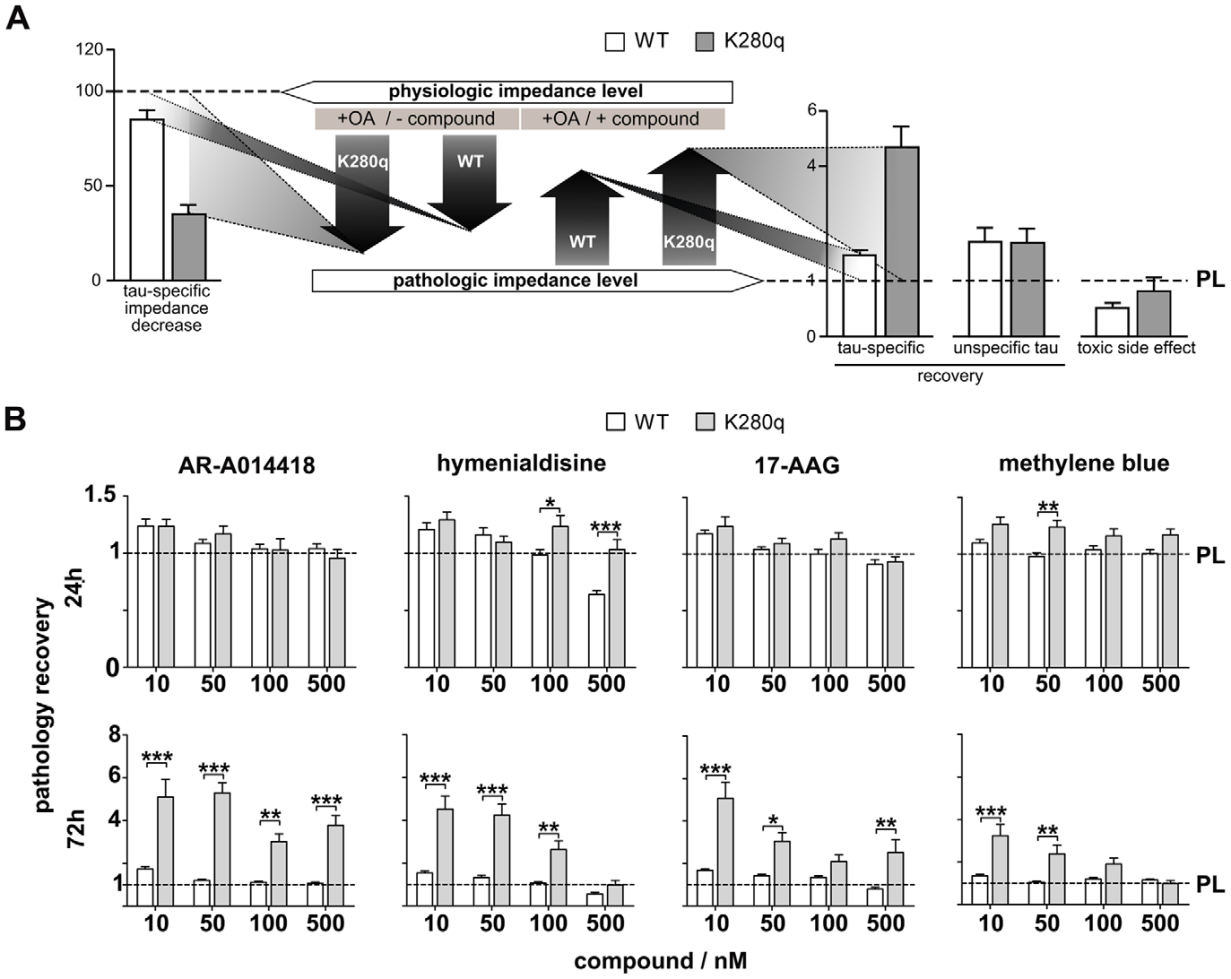
Impedimetric quantification of compound efficacy for tau-specific neurodegeneration attenuation and detection of toxic side effects. **A.** Scheme of impedimetric detection of tau-specific neurodegeneration by using our WT and K280q expressing SH-SY5Y 3D culture model. The induction of tau pathology by OA reduces the physiological impedance level (100% normalization for the untreated tau variants) in a tau mutant manner leading to the pathologic impedance level for WT and K280q tau. Based on this OA induced tau-specific impedance decrease, compounds can be analyzed for their ability to recover induced pathology. For quantitative comparison, all values are normalized to the pathological level of 25 nM OA treated cultures (PL set to 1.0). The Comparison of WT and K280q recovery values allow quantitative distinction of tau-specific effects (recovery level K280q > WT ≥1), common cellular recovery (recovery level WT ≥ K280q ≥1) and potential toxic side effects (recovery level ≤1). **B.** Impedimetric analysis of the reference compounds AR-A014418 and hymenialdisine (tau kinase inhibitors), 17-AAG (HSP-90 inhibitor) and methylene blue (aggregation inhibitor). The pathology recovery (x-fold change to the pathology level, PL) of a 24 h and 72 h treatment of spheroids with 25 nM OA and the reference compounds compared in comparison to cultures treated only with 25 nM OA. (n = 20 *p<0.05, **p<0.01, ***p<0.001).

Altogether, we were able to detect and quantify mutation number- and therewith tau-specific neurodegeneration in a time- and OA concentration-dependent manner with high sensitivity and precision. With the help of apoptosis marker expression analysis and live cell imaging, we were able to distinguish impedimetrically between the retraction of neurites as one essential pathological hallmark of AD and massive cell death that occurred at OA concentrations higher than 25 nM. Thus, comparing apoptosis marker expression and imaging results with the impedimetric analysis, the latter is able to detect pathological alterations much earlier and more specific. Moreover, the increasing and therefore tau-specific differences across the WT tau expressing 3D-cultures and our novel high-pathologic fourfold mutated tau K280q expressing 3D-cultures combined with our automatable impedimetric monitoring system offer a perfect platform for high content compound testing (Fig. 4A). In this context, we identified an OA concentration of 25 nM as most convenient for a suitable pathology monitoring window between treated WT and K280q cultures that represents the tau-specific effect.

### Impedance Spectroscopy-based Quantification of Tau-specific Compound Efficacy

To demonstrate the capability of our developed system for compound efficacy quantification, we chose four different reference compounds focusing on different tau pathology-related targets and clearing mechanisms, respectively. Therefore, untreated WT and K280q tau expressing spheroids were used that differed in the expression of a non-mutated and mutant tau variant and were characterized by a physiological impedance level (normalized to 100%). To determine the disease recovery potential of the described tau-specific reference compounds, spheroids were treated with 25 nM OA in the absence (defined as pathological impedance level, PL) or presence (defined as pathology recovery) of compounds at different concentrations (10–500 nM) for 24 and 72 hours. Measurements were aimed to identify whether there was a tau-specific pathology recovery (different recovery values for WT and K280q ≥1), a more common cellular pathology recovery (similar recovery pattern for WT and K280q ≥1) or even a toxic side effect (declined recovery values for WT and K280q ≤1) of the compound (Fig. 4A). All values were expressed as x-fold changes compared to the appropriate OA-treated spheroids (25 nM) that represent the pathological level (PL normalized to 1.0). With the aim to identify tau pathology-specific compounds, a higher, most likely significant recovery for K280q is desired.

The values for 24 hours and 72 hours treatment (Fig. 4B) as well as relative cross section areas of spheroids were determined (Fig. S1). After 24 hours treatment, only few significant impedance differences between WT and K280q tau spheroids were observed. The relative cross section area analysis also revealed only few significant differences between the two tau variants with WT tau spheroids always showing the higher cross section area. Extreme significant effects were detected after 72 hours incubation. Even small concentrations of the compounds led to three to five times higher recovery values in K280q tau expressing spheroids compared to the WT tau expressing spheroids. Highest tau-specific pathology recovery was observed for 10 nM AR-A014418 (WT: 1.73±0.12, K280q: 5.10±0.82) and 17-AAG (WT: 1.67±0.07, K280q: 5.07±0.75) followed by hymenialdisine (WT: 1.54±0.09, K280q: 4.52±0.61) and methylene blue (WT: 1.35±0.07, K280q: 3.24±0.53). In contrast, at higher concentrations (500 nM) of hymenialdisine and 17-AAG values below the pathologic level were detected especially in WT tau expressing spheroids (0.55±0.08 and 0.79±0.08), which may indicate toxic side effects. In contrast, high concentrations of the specific GSK-3 inhibitor AR-A014418 did not cause toxic side effects (WT: 1.06±0.06). Moreover, the analysis of the cross section area could provide additional information for a comprehensive view on compound effects (Fig. S1). Treatment with AR-A014418, hymenialdisine and 17-AAG for 72 hours led to an increased cross section area compared to the pure OA-treated samples in both tau variants. In contrast, the incubation with methylene blue provoked recovery values that were always lower than one. However, only few significant cross section area differences between WT and K280q tau spheroids were detected.

These findings demonstrate that even small concentrations in the low nanomolar range of the reference compounds led to strongly increased recovery values after prolonged incubation for 72 hours. Furthermore, effects were found to be tau mutation-dependent, since they were much more intensive in the high-pathologic K280q tau expressing 3D cultures than in WT tau expressing 3D cultures. Furthermore, there were hints that high concentrations of especially hymenialdisine and methylene blue caused cytotoxicity, whereas particularly AR-A014418 showed protective effects over a wide concentration range. However, the spheroid cross section area was not intensively affected by tau-focused therapeutic treatment indicating the high sensitivity of our impedance screening system to detect cellular recovery processes.

## Discussion

Despite all efforts for development and establishment of especially tauopathy *in vivo* models [14], there is a high demand for comprehensive *in vitro* models. These have to fulfill the prerequisites to recapitulate the complex AD-related taupathological mechanism and the demands for standardization, availability and reproducibility. Especially in the field of API development, these demands could not be sufficiently fulfilled until today. In this context, we established a novel 3D tau pathology culture model. In combination with our sensitive impedance spectroscopy-based label-free monitoring system, it could bridge the gap between insufficient artificial 2D cell culture assays [17], [31], [33], [34] and time-consuming and hardly up scalable *in vivo* models [14], [35]. Using SH-SY5Y cells, we avoid restrictions of primary cells or even stem cell-derived neurons with regard to availability, reproducibility and time. To overcome limitations of monolayer culture based SH-SY5Y neurodegenerative model systems [17], [31], [36], we established a 3D culture system, in which we could essentially improve the neuronal characteristics of the cells by an extended autonomous differentiation simply replacing the common 2D culture by a 3D cell structure system without the need for any artificial differentiating agents. This is particularly important for the native and unaffected induction of tau pathological processes, since widely used artificial differentiating agents like staurosporine and retinoic acid that are necessary for 2D culture models perturb several pathways involved in AD-like pathology progression [29], [37]. Overall, based on their three-dimensional cell organization, spheroids are more suitable to study organotypic properties *in vitro* [38].

Moreover, we established a novel fourfold mutated tau variant (K280q) that showed a drastically increased pathology in comparison to widely used wildtype tau (WT), single mutated tau (P301L, ΔK280) or even threefold tau variant [14], [32], [39]. In contrast to previous OA-based tau hyperphosphorylation pathology models [8], [17], our novel system provides a very fast and intensified pathology development. Moreover, the comparative analysis of the WT and K280q tau cells allows detailed quantitative information for tau-specific degenerative processes (Fig. 4A).

**Figure 5:**
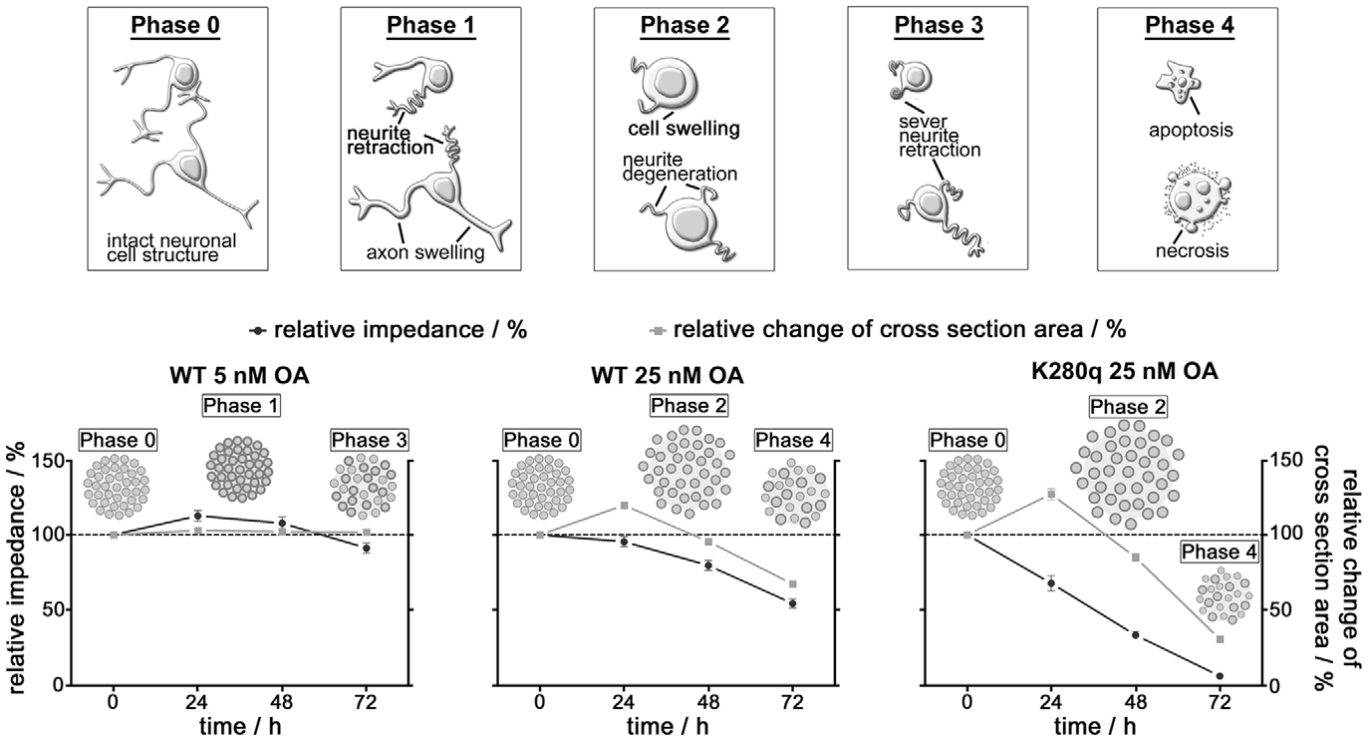
Scheme of tau mutation dependent cellular neurodegeneration mechanism based on observed relative impedance and cross section area alteration monitoring. Combination of time-dependent relative impedance (dark grey circles) and cross section area (light grey quad) alterations lead to a model of spheroid degradation that is in line with the obtained molecular and microscopic data. Comparing wildtype tau expressing spheroids incubated with 5 nM and 25 nM OA, hyperphosphorylation leads to an extended degeneration. More strikingly, comparison of wildtype tau (WT) and K280q tau (K280q) expressing spheroids reveals the tau-specific neurodegenerative processes.

A more detailed understanding of the underlying tau pathological degenerative processes could be obtained combining the analysis of spheroid impedance spectroscopy and cross section area (Fig. 5). Interestingly, especially for the WT tau expressing spheroids low OA concentrations led to a relative impedance increase coexistent with a constant cross section area, whereas higher OA concentrations caused a cross section area increase accompanied by strongly decreased relative impedance. Recently, it has been shown that AD pathology induces a continuing process of axon sprouting and swelling in neurons leading to increased membrane and decreased electrolyte properties [40], [41] assuming similar effects that could be detected after 24 hours incubation with 5 nM OA. Subsequently, the retraction of neuronal processes is initiated by microtubule instability leading to the breakdown of the neuronal network and cell disintegration of spheroidal structure [42] determined after 72 hours OA treatment. In contrast, accelerated cell damage is characteristic at higher OA concentrations of 25 nM. Furthermore, swelling of the neuronal cell body could be visualized via microscopic images of SH-SY5Y spheroids after 24 hours incubation. Cell swelling is characteristic for accelerated neurodegeneration leading to necrosis/apoptosis, which were found to coexist in AD patient brains [43]. Despite the cellular swelling, a continuous impedance decrease was observed. This can be correlated to extended degeneration processes that accompany loss of cell-cell-/cell-matrix-contacts and cause therewith an increased intercellular volume. Finally, progressive cell loss also induces spheroid size reduction and a strong proceeding of conductive elements that evoke further impedance decrease after 72 hours treatment with 25 nM OA. Herewith, we could show that the distinction of neurite retraction versus massive cell death and therewith the selective detection of tau-specific degeneration and regeneration is possible using our impedance-based screening system in combination with optical monitoring without the need for further molecular end point analysis. Furthermore, our results reveal evidences for processes, which are highly analogue to *in vivo*-like explant culture models [15], [40] demonstrating the *in vivo*-like complex character of our 3D culture model.

More strikingly, we were able for the first time to sensitively detect the attenuating effect of the potential API candidates AR-A014418, hymenialdisine, 17-AAG and methylene blue against OA-induced tauopathy in the low nanomolar concentration range. Especially the very selective GSK-3β inhibitor AR-A014418 performed very well when considering potential cytotoxic side effects that could be detected simultaneously. Taken together, we demonstrate that our novel 3D tauopathy culture model in combination with our impedance spectroscopy-based label-free monitoring system is suitable for high content compound testing. Since our system uses single spheroids that are individually traced and analyzed over time with only 3000 to 5000 cells per spheroid the bioelectronic-based read-out fulfills the demands of modern high-content screening systems regarding cell content and material efforts. Therefore, our neuronal 3D *in vitro* model is positioned between simple 2D culture and complex, cost and time consuming animal models and is therefore suitable to improve the evaluation of APIs in preclinical trials. Moreover, the established 3D *in vitro* model can be easily modified and extended to investigate further aspects like induction of taupathological consequences (e.g. toxic β-amyloid species or shift from 3R to 4R tau isoforms). Furthermore, the established cell culture model is highly appropriate for further applications in the field of neurodegenerative diseases (e.g. patient-specific induced pluripotent stem cells offering a platform for personalized medicine) [5].

### Conclusions

In conclusion, the presented results show the high suitability and advantages of our novel neuronal 3D *in vitro* tauopathy cell culture model. In combination with our microcavity array-based label-free monitoring system we successfully demonstrated the quantitative monitoring of tau-specific neurodegenerative processes in individual 3D-cultures and linking them to distinct pathological mechanisms. More strikingly, we demonstrate the capability of our system for compound efficacy as well as side effect testing. This makes our 3D model system suitable for high content screening systems especially in the focus of API development against AD and related tauopathies and a valid alternative model to 2D cell culture and complex animal models.

## Materials and Methods

### SH-SY5Y Cell Cultivation, Spheroid Formation and Okadaic Acid-induced Tau Hyperphosphorylation

SH-SY5Y cells (ATCC) were cultured in DMEM medium supplemented with 15% fetal bovine serum, 1% non-essential amino acids, 2 mM glutamax, 100 units/ml streptomycin/penicillin and 0.4% gentamicine (Invitrogen) at 37°C in a humidified atmosphere with 5% CO_2_. Spheroids were generated by seeding 2 million cells per well in 6-well plates and cultivation on a self-developed gyratory shaker (72 rpm).

Tau hyperphosphorylation was induced by the application of okadaic acid (Sigma-Aldrich) in a concentration range from 5–100 nM. Reference compounds AR-A014418 (Sigma-Aldrich), 17-allylamino-17-demothoxygeldanamycin (17-AAG) (Sigma-Aldrich), hymenialdisine (Enzo LifeScience) and methylene blue (Sigma-Aldrich) were used in a concentration range from 10–500 nM. For individual tracking, at culture day four single SH-SY5Y spheroids were transferred into a single well of a 48-well plate and experiments were performed at culture day five.

### Generation of SH-SY5Ycell Lines Stably Expressing EGFP-fused Tau Constructs by Lentiviral Transduction

Human wildtype tau (0N4R) cDNA was amplified by PCR from isolated human neuronal cell mRNA and cloned into the pEGFP-C1 vector C-terminal to the EGFP coding sequence. For generating replication incompetent lentiviral particles the EGFP-tau wildtype coding sequence was cloned into the pLenti6v5-topo vector (Invitrogen). Single mutated (P301L) and fourfold mutated K280q (ΔK280, P301L, V337M, R406W) variants were obtained by site-directed mutagenesis (QuickChange Lightning, Stratagene). Virus particles were generated in accordance to the manufactures guide (Lentiviral TOPO Expression Kit, Invitrogen) and SH-SY5 cells were transduced and selected by incorporated blasticidine (Invitrogen) resistance (10 µg/ml).

### Protein Chemical Analysis

For protein extraction, spheroids were transferred in RIPA-buffer supplemented with protease and phosphatase inhibitor cocktail (Sigma-Aldrich) and sonificated (Hielscher GmbH). The protein concentration was determined using the Roti-Nanoquant Assay (Carl-Roth GmbH). 25 µg protein was treated with Laemmli sample buffer, separated on a 10% SDS polyacrylamide gel and blotted on a PVDF membrane applying 2 mA/cm^2^. Immunolabeling was realized using specific primary antibodies (p-tau_T212_, p-tau_S422_, p-tau_S262_, all three form Merck (1∶1000); anti-neurofilament-L, SantaCruz, 1∶1000; anti-β III-tubulin, SantaCruz, 1∶1000; anti-synapsin I, Covance, 1∶1000; anti-cleaved PARP [Asp214], BD Pharming, 1∶1000; anti-caspase 3–cleaved tau [Asp421], SantaCruz, 1∶1000) at 4°C overnight. Specific protein signals were detected applying a horseradish peroxidase-conjugated secondary antibodys (Dianova, 1∶5000) via a chemiluminescence detection kit (MobiTec) and the ChemiDoc-XRS documentation system (BioRad). Membranes were stripped and reloaded with anti-tau human (Dako, 1∶1000) and β-tubulin (Abcam, 1∶1000), respectively to perform densitometric quantification using QuantityOne Software v4.6 (BioRad).

### Immunocytochemical Staining

For cryodissection spheroids were fixed with 1 ml formaldehyde (4%) for one hour and transferred to 1 ml saccharose (25%) for several days. Spheroids were cryodissected (slices with a thickness of 20 µm) with a freeze microtome (Leica, CM3050S) and stored at −20°C. For immunocytochemistry, the slices were blocked with 3% BSA/0.1% Triton-X in PBS for 45 min and incubated with primary antibody (anti-neurofilament-200, SantaCruz, 1∶500; p-tau_S262_, Merck, 1∶500; anti-MC-1, kindly provided by Prof. Dr. Peter Davies from the Albert Einstein College of Medicine, 1∶50) in blocking solution for two hours at room temperature. After three washing steps with PBS, the slices were treated with secondary antibody (Dylight-549, Dianova, 1∶100) in blocking solution for one and a half hours at room temperature. Nuclei were stained with DAPI (Sigma, 1 µg/ml). Images were taken by laser scanning microscopy.

### Life Cell Imaging of SH-SY5Y 3D Cultures Expressing EGFP-fused Tau via Laser Scanning Confocal Microscopy

Microscopic images of SH-SY5Y cells expressing EGFP-tau fusion protein were obtained by an inverse Nikon TE2000 microscope coupled with a Nikon digital eclipse C1plus confocal laser scanning module and the EZ-C1 3.1 Nikon spectral imaging software. EGFP was excited with the 488 nm Argon laser and detected with the 515±15 nm band pass filter. All images were deconvoluted using 3D Huygens Deconvolution & Analysis Software v3.6 (SVI) and presented at maximum intensity projections.

### Determination of Spheroid Morphology and Cross Section Area

Microscopic images of individual SH-SY5Y spheroids were taken every 24 hours after impedimetric measurement using a Nikon Eclipse TE2000U microscope. Spheroid cross section area was determined with the software ImageJ (National Institute of Health, USA) by manually marking spheroid contour.

### Electrical Impedance Spectroscopy

Impedimetric measurements were performed as former described [16]. Briefly, five days old spheroids were transferred to a self-developed microcavity array (MCA) with pyramidal cavities (edge length 200 µm) comprising of one electrode on each of the four cavity walls. The MCA was equilibrated with phosphate buffered saline (Invitrogen) and positioned in a self-developed, automated measurement system based on the high precision impedance analyzer Agilent 4294A (Agilent Technologies). Impedance spectra were recorded in a frequency interval ranging from 5 kHz to 5 MHz applying an alternating voltage of 100 mV. The self-developed software IMAT v1.8g (Impedance Measurement and Automation Tool) and IDAT v.3.6 (Impedance Data Analyzing Tool) were used for instrument controlling, data recording and processing. Cellular contribution to the impedance magnitude was extracted by automated calculation of the relative impedance (|Z|_with spheroid_−|Z|_without spheroid_)/|Z|_without spheroid_ × 100%). For every measurement on a spheroid, six spectra were automatically acquired (each electrode measured against each other) and averaged by the software resulting in reproducible and positioning independent cell signal. Furthermore, the maximum contribution (peak) was determined by the IDAT v3.6 software. For statistical analysis the relative impedance maxima were normalized to the experimental start (0 h).

### Statistical Analysis

Statistical analysis was done using Prism 5 (GraphPad Software). All values are presented as means ± standard error of mean (s.e.m.) unless described differently. For all presented graphs, complete values (means and s.e.m. or s.d.) are listed in tables in the dataset S1. Multiple group comparison was done using two-way ANOVA and Bonferroni post hoc test. Differences between two means were considered significant with *p<0.05, very significant with **p<0.01 and extreme significant with ***p<0.001.

## Supporting Information

Figure S1 **Cross section area analysis of pathology recovery by reference compounds.**
(TIF)

Movie S1 **Reconstructed and animated z-stack of an EGFP-fused WT tau expressing spheroid live image at day five.**
(AVI)

Dataset S1 **Complete values including s.e.m. or s.d. that are presented in the graphs.**
(RTF)
