# Supplementary figures and images for: Induced Tauopathy in a Novel 3D-Culture Model Mediates Neurodegenerative Processes: A Real-Time Study on Biochips
Source: PLoS One. 2012 Nov 7;7(11):e49150. doi: 10.1371/journal.pone.0049150 (PMC3492324; doi:10.1371/journal.pone.0049150)

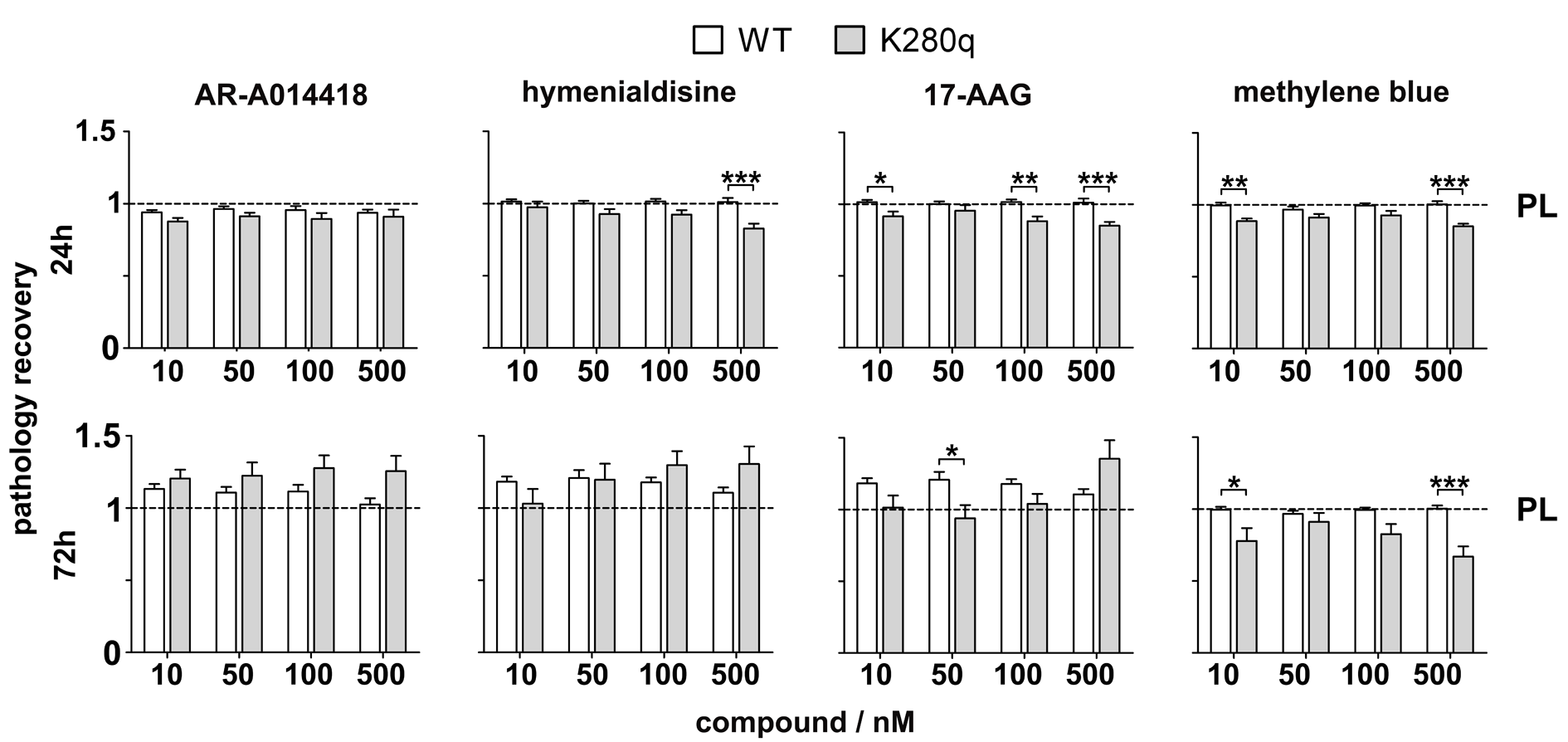

Supplement: Figure S1 — Cross section area analysis of pathology recovery by reference compounds. (TIF) [file pone.0049150.s001.tif]
