# Supplementary material for: Induced Tauopathy in a Novel 3D-Culture Model Mediates Neurodegenerative Processes: A Real-Time Study on Biochips
Source: PLoS One. 2012 Nov 7;7(11):e49150. doi: 10.1371/journal.pone.0049150 (PMC3492324; doi:10.1371/journal.pone.0049150)
Supplement: Dataset S1 — Complete values including s.e.m. or s.d. that are presented in the graphs. (RTF) [file pone.0049150.s003.rtf]

Additional File 3

Table S1 – SH-SY5Y spheroid size development over culture days (DIV). Expressed as diameter (mean ± s.d., n = 500).
DIV	1	2	3	4	5	6	7	
WT	65.3 ± 13.9	122.0 ± 20.5	141.2 ± 23.8	146.5 ± 20.6	159.7 ± 25.5	172.5 ± 29.0	181.5 ± 31.4	
P301L	66.3 ± 15.0	94.8 ± 20.6	116.5 ± 22.5	125.1 ± 24.2	148.0 ± 30.7	156.5 ± 28.5	163.6 ± 30.1	
K280q	86.0 ± 31.7	107.1 ± 27.3	123.5 ± 32.6	139.8 ± 29.5	154.6 ± 35.8	162.1 ± 26.5	160.2 ± 34.4	


Table S2 - Protein expression analysis of OA treated SH-SY5Y spheroids. (mean ± s.e.m., n = 3)
		control	10 nM OA	25 nM OA	50 nM OA	100 nM OA	
p-tauT212	WT	25.9 ± 1.2	36.9 ± 8.3	23.7 ± 1.4	34.2 ± 5.2	37.8 ± 6.0	
	P301L	17.2 ± 4.4	22.0 ± 4.2	30.6 ± 6.7	59.7 ± 6.5	77.1 ± 26.4	
	K280q	20.6 ± 5.3	25.0 ± 0.2	31.2 ± 8.8	41.0 ± 7.0	57.5 ± 12.9	
p-tauS262	WT	59.6 ± 12.4	55.6 ± 14.4	60.6 ± 8.4	61.7 ± 4.0	72.0 ± 4.3	
	P301L	54.5 ± 8.0	50.9 ± 8.4	58.7 ± 9.2	80.3 ± 8.9	106.8 ± 3.5	
	K280q	54.0 ± 16.3	61.0 ± 13.4	56.1 ± 9.0	81.6 ± 8.5	91.3 ± 21.2	
p-tauS422	WT	7.7 ± 4.5	12.1 ± 4.6	7.1 ± 1.9	10.4 ± 4.4	37.6 ± 14,8	
	P301L	14.2 ± 2.8	64.1 ± 21.1	53.4 ± 9.3	48.3 ± 18.2	39.0 ± 18.3	
	K280q	27.8 ± 8.5	85.9 ± 18.0	64.9 ± 21.5	62.8 ± 13.8	58.3 ± 23.1	
NF-L	WT	62.1 ± 5.3	71.1 ± 9.5	84.0 ± 10.2	54.4 ± 15.3	32.6 ± 17.6	
	P301L	65.6 ± 9.6	75.7 ± 12.9	45.9 ± 8.3	22.0 ± 6.9	12.3 ± 4.6	
	K280q	59.3 ± 8.0	77.9 ± 4.9	62.0 ± 9.0	34.9 ± 6.9	20.2 ± 5.1	
cl-tau	WT	1.8 ± 1.2	1.7 ± 0.2	15.5 ± 11.1	13.0 ± 8.2	9.1 ± 2.6	
	P301L	2.9 ± 0.5	5.8 ± 2.4	55.3 ± 11.8	91.1 ± 13.4	74.6 ± 10.1	
	K280q	4.3 ± 2.0	20.6 ± 6.7	72.8 ± 7.5	85.9 ± 7.2	76.9 ± 17.0	
cl-PARP	WT	3.6 ± 0.9	4.4 ± 1.4	46.4 ± 7.9	71.7 ± 11.6	63.0 ± 14.1	
	P301L	15.5 ± 2.1	13.1 ± 4.1	71.3 ± 10.9	95.7 ± 20.8	85.5 ± 13.8	
	K280q	7.8 ± 1.2	8.6 ± 4.4	85.1 ± 8.5	98.9 ± 20.1	91.0 ± 30.7	


Table S3 – Impedimetric analysis of OA treated SH-SY5Y spheroids. (mean ± s.e.m., n = 30)
		5 nM OA	10 nM OA	25 nM OA	100 nM OA	
24 h	WT	112.7 ± 3.7	116.1 ± 2.1	95.4 ± 3.5	48.0 ± 4.1	
	P301L	103.0 ± 2.7	84.3 ± 4.0	79.4 ± 2.7	44.8 ± 2.9	
	K280q	97.8 ± 2.7	100.2 ± 4.0	67.7 ± 5.1	26.2 ± 3.6	
48 h	WT	107,8 ± 4.1	107.5 ± 4.0	79.6 ± 3.3	17.0 ± 1.9	
	P301L	92.5 ± 3.2	77.8 ± 2.6	52.5 ± 3.6	16.9 ± 2.4	
	K280q	92,2 ± 4.0	85.9 ± 3.1	33.2 ± 2.3	6.3 ± 1.0	
72 h	WT	91.2 ± 3.4	89.9 ± 3.5	54,2 ± 3.0	7.2 ± 2.0	
	P301L	86,7 ± 3.3	70.7 ± 1.8	16.5 ± 2.1	9.9 ± 1.6	
	K280q	80.9 ± 3.4	52.2 ± 2.8	5.8 ± 1.5	2.4 ± 0.6	

Table S4 – Cross section area analysis of OA treated SH-SY5Y spheroids.(mean ± s.e.m., n = 30)
		5 nM OA	10 nM OA	25 nM OA	100 nM OA	
24 h	WT	102.9 ± 1.2	103.4 ± 1.2	119.7 ± 1.9	99.8 ± 2.8	
	P301L	108.9 ± 1.7	118.2 ± 2.7	133.7 ± 3.9	105.1 ± 3.3	
	K280q	110.7 ± 2.1	116.9 ± 2.1	127.1 ± 3.8	70.7 ± 4.1	
48 h	WT	102.1 ± 1.1	106.5 ± 1.7	95.5 ± 1.3	82.2 ± 2.3	
	P301L	109.3 ± 2.2	126.0 ± 3.9	116.1 ± 3.5	59.0 ± 3.4	
	K280q	110.7 ± 2.1	118.9 ± 2.2	85.0 ± 2.6	36.1 ± 3.1	
72 h	WT	101.7 ± 1.4	85.4 ± 2.3	67.3 ± 1.9	59.2 ± 2.6	
	P301L	114.3 ± 2.7	94.6 ± 3.6	48.2 ± 3.1	34.0 ± 3.1	
	K280q	100.0 ± 2.2	103.4 ± 1.2	30.5 ± 1.6	19.9 ± 1,7	


Table S5 – Imepdimetric analysis of pathology recovery after compound treatment. (mean ± s.e.m., n = 20)

		10 nM	50nM	100 nM	500 nM	
		24 h	72 h	24 h	72 h	24 h	72 h	24 h	72 h	
AR-A014418	WT	1.23 ± 0.06	1.73 ± 0.12	1.09 ± 0.03	1.20 ± 0.06	1.04 ± 0.04	1.12 ± 0.05	1.04 ± 0.04	1.06 ± 0.06	
	K280q	1.23 ± 0.06	5.10 ± 0.82	1.17 ± 0.07	5.29 ± 0.47	1.03 ± 0.1	3.01 ± 0.37	95.7 ± 7.7	3.78 ± 0.46	
hymenial-disine	WT	1.21 ± 0.06	1.54 ± 0.09	1.16 ± 0.06	1.32 ± 0.10	0.95 ± 0.05	1.07 ± 0.06	0.64 ± 0.04	0.55 ± 0.08	
	K280q	1.29 ± 0.07	4.53 ± 0.61	1.10 ± 0.05	4.24 ± 0.53	1.23 ± 0.1	2.63 ± 0.41	1.03 ± 0.09	0.98 ± 0.21	
17-AAG	WT	1.18 ± 0.03	1.67 ± 0.07	1.04 ± 0.02	1.42 ± 0.07	1.00 ± 0.04	1.34 ± 0.08	0.91 ± 0.04	0.80 ± 0.08	
	K280q	1.24 ± 0.09	5.07 ± 0.75	1.09 ± 0.05	3.04 ± 0.41	1.13 ± 0.06	2.09 ± 0.32	0.93 ± 0.04	2.52 ± 0.61	
methylene blue	WT	1.10 ± 0.03	1.35 ± 0.08	0.98 ± 0.03	1.05 ± 0.05	1.04 ± 0.04	1.21 ± 0.06	1.00 ± 0.03	1.15 ± 0.06	
	K280q	1.26 ± 0.06	3.24 ± 0.53	1.24 ± 0.06	2.38 ± 0.41	1.16 ± 0.06	1.91 ± 0.28	1.17 ± 0.05	0.98 ± 0.15	


Table S6 – Chross section area analysis of pathology recovery after compound treatment. (mean ± s.e.m., n = 20)

		10 nM	50nM	100 nM	500 nM	
		24 h	72 h	24 h	72 h	24 h	72 h	24 h	72 h	
AR-A014418	WT	0.94 ± 0.02	1.13 ± 0.03	0.94 ± 0.02	1.11 ± 0.04	0.96 ± 0.03	1.11 ± 0.05	0.94 ± 0.02	1.03 ± 0.04	
	K280q	0.88 ± 0.03	1.21 ± 0.06	0.91 ± 0.03	1.22 ± 0.09	0,90 ± 0.04	1.28 ± 0.09	0.91 ± 0.05	1.26 ± 0.11	
hymenial-disine	WT	1.01 ± 0.02	1.18 ± 0.04	1.00 ± 0.02	1.21 ± 0.06	1.02 ± 0.02	1.18 ± 0.03	1.01 ± 0.03	1.11 ± 0.04	
	K280q	0.97 ± 0.04	1.03 ± 0.10	0.93 ± 0.03	1.20 ± 0.11	0.92 ± 0.03	1.30 ± 0.10	0.83 ± 0.03	1.31 ± 0.12	
17-AAG	WT	1.01 ± 0.02	1.18 ± 0.04	1.00 ± 0.02	1.21 ± 0.06	1.02 ± 0,02	1.18 ± 0,03	1.01 ± 0.03	1.11 ± 0.04	
	K280q	0.92 ± 0.03	1.01 ± 0.08	0.95 ± 0.04	0.94 ± 0.09	0.88 ± 0.03	1.04 ± 0.07	0.85 ± 0.03	1.35 ± 0.13	
methylene blue	WT	1.00 ± 0.02	1.00 ± 0.02	0.97 ± 0.02	0.97 ± 0.02	0.99 ± 0.02	0.99 ± 0.02	1.00 ± 0.02	1.00 ± 0.2	
	K280q	0.89 ± 0.02	0.78 ± 0.09	0.91 ± 0.02	0.91 ± 0.06	0.93 ± 0.03	0.83 ± 0.07	0.85 ± 0.02	0.67 ± 0.07	
